# Relationship Between Lipoprotein(a), Renal Function Indicators, and Chronic Kidney Disease: Evidence From a Large Prospective Cohort Study

**DOI:** 10.2196/50415

**Published:** 2024-01-31

**Authors:** Yingxin Liu, Ruoting Wang, Shuai Li, Changfa Zhang, Gregory Y H Lip, Lehana Thabane, Guowei Li

**Affiliations:** 1 Center for Clinical Epidemiology and Methodology, Guangdong Second Provincial General Hospital Guangzhou China; 2 Liverpool Centre for Cardiovascular Science at University of Liverpool, Liverpool John Moores University and Liverpool Heart & Chest Hospital Liverpool United Kingdom; 3 Danish Center for Health Services Research, Department of Clinical Medicine, Aalborg University Aalborg Denmark; 4 Department of Health Research Methods, Evidence, and Impact, McMaster University Hamilton, ON Canada; 5 Father Sean O’Sullivan Research Centre, St Joseph’s Healthcare Hamilton Hamilton, ON Canada

**Keywords:** lipoprotein(a), chronic kidney disease, renal function, urinary albumin-to-creatinine ratio, glomerular filtration rate

## Abstract

**Background:**

Chronic kidney disease (CKD) poses a significant global public health challenge. While lipoprotein(a) (Lp[a]) has been established as a significant factor in cardiovascular disease, its connection to CKD risk remains a topic of debate. Existing evidence indicates diverse risks of kidney disease among individuals with various renal function indicators, even when within the normal range.

**Objective:**

This study aims to investigate the joint associations between different renal function indicators and Lp(a) regarding the risks of incident CKD in the general population.

**Methods:**

The analysis involved a cohort of 329,415 participants without prior CKD who were enrolled in the UK Biobank between 2006 and 2010. The participants, with an average age of 56 (SD 8.1) years, included 154,298/329,415 (46.84%) males. At baseline, Lp(a) levels were measured using an immunoturbidimetric assay and classified into 2 groups: low (<75 nmol/L) and high (≥75 nmol/L). To assess participants’ baseline renal function, we used the baseline urine albumin-to-creatinine ratio (UACR) and estimated glomerular filtration rate (eGFR). The relationship between Lp(a), renal function indicators, and the risk of CKD was evaluated using multivariable Cox regression models. These models were adjusted for various factors, including sociodemographic variables, lifestyle factors, comorbidities, and laboratory measures.

**Results:**

A total of 6003 incident CKD events were documented over a median follow-up period of 12.5 years. The association between elevated Lp(a) levels and CKD risk did not achieve statistical significance among all participants, with a hazard ratio (HR) of 1.05 and a 95% CI ranging from 0.98 to 1.13 (*P*=.16). However, a notable interaction was identified between Lp(a) and UACR in relation to CKD risk (*P* for interaction=.04), whereas no significant interaction was observed between Lp(a) and eGFR (*P* for interaction=.96). When compared with the reference group with low Lp(a) and low-normal UACR (<10 mg/g), the group with high Lp(a) and low-normal UACR exhibited a nonsignificant association with CKD risk (HR 0.98, 95% CI 0.90-1.08; *P*=.74). By contrast, both the low Lp(a) and high-normal UACR (≥10 mg/g) group (HR 1.16, 95% CI 1.08-1.24; *P*<.001) and the high Lp(a) and high-normal UACR group (HR 1.32, 95% CI 1.19-1.46; *P*<.001) demonstrated significant associations with increased CKD risks. In individuals with high-normal UACR, elevated Lp(a) was linked to a significant increase in CKD risk, with an HR of 1.14 and a 95% CI ranging from 1.03 to 1.26 (*P*=.01). Subgroup analyses and sensitivity analyses consistently produced results that were largely in line with the main findings.

**Conclusions:**

The analysis revealed a significant interaction between Lp(a) and UACR in relation to CKD risk. This implies that Lp(a) may act as a risk factor for CKD even when considering UACR. Our findings have the potential to provide valuable insights into the assessment and prevention of CKD, emphasizing the combined impact of Lp(a) and UACR from a public health perspective within the general population. This could contribute to enhancing public awareness regarding the management of Lp(a) for the prevention of CKD.

## Introduction

Chronic kidney disease (CKD), a significant contributor to cardiovascular disease (CVD) and mortality, affected approximately 697.5 million individuals worldwide, with a prevalence of 9.1% in 2017 [1,2]. In public health and clinical research, CKD is typically diagnosed using the estimated glomerular filtration rate (eGFR) and urine tests to detect the presence of albumin or protein or a combination of both [3]. As per recommendations [3], a diagnosis of CKD can be made when the eGFR is <60 mL/min/1.73 m^2^ and is combined with albuminuria, usually defined as a urinary albumin-to-creatinine ratio (UACR) ≥30 mg/g.

Lipoprotein(a) (Lp[a]) is a particle resembling low-density lipoprotein (LDL), comprising a large glycoprotein apolipoprotein(a) bound to an apolipoprotein B100 molecule [4]. The adverse impact of Lp(a) on CVD has been robustly substantiated by epidemiological, experimental, and genetic studies [5,6]. By contrast, the association between Lp(a) and the risk of CKD is still a topic of debate, with inconsistent findings reported in both observational studies and Mendelian randomization studies [7-14]. As a result, Lp(a) has not yet been incorporated into current guidelines or public health policies for CKD prevention, detection, management, or surveillance [3,15]. A deeper understanding of the role of Lp(a) in CKD could enhance public initiatives for the prevention and risk management of CKD. Therefore, further explorations are needed to investigate the relationship between Lp(a) and CKD risk.

Remarkably, no previous studies have investigated the association between the risk of CKD and Lp(a) in conjunction with eGFR or UACR, despite both measures being commonly used as indicators of renal function [8-10]. Heterogeneous risks of CKD have been observed in participants with different baseline eGFR or UACR measures, even when these 2 indicators were within the normal range [16-19]. For example, evidence has suggested that a UACR value in the high-normal range (10-30 mg/g) is significantly associated with the progression of CKD and renal failure when compared with the low-normal group (<10 mg/g) used as a reference [17,18,20]. While some previous studies generally adjusted for renal function indicators in their regression models, the association between Lp(a) and CKD risk was estimated based on an average level of renal function indicators. Therefore, the inconsistent associations between Lp(a) and CKD risk may, at least in part, depend on participants’ varying renal function levels across different studies.

In this study, our objective was to investigate the combined associations of Lp(a) and renal function indicators in relation to CKD risk among participants without a history of CKD from the UK Biobank cohort study. Exploring the potential interplay between Lp(a) and renal function may provide new evidence for assessing and preventing CKD risk in the general population from a public health perspective. This research could contribute to raising public awareness about the importance of managing Lp(a) for CKD prevention.

## Methods

### Study Population

Information about the UK Biobank study has been extensively documented in prior literature and is available on the official website [21,22]. In summary, the UK Biobank is a comprehensive cohort study that encompasses biological and medical data from approximately half a million residents in the United Kingdom since 2006. Enrolled participants provided written informed consent, and the study received approval from the North West Multi-Centre Research Ethics Committee.

A total of 502,411 participants were included in our analyses. Exclusions were made for participants with missing data on eGFR (n=33,141), UACR (n=13,408), or Lp(a) (n=81,863), as well as those with renal dysfunction at baseline, including a diagnosis of CKD, an eGFR<60 mL/min/1.73 m^2^, or a UACR≥30 mg/g (n=44,584). Consequently, the final analysis included 329,415 participants. The participant selection process is depicted in Figure S1 in Multimedia Appendix 1.

### Outcomes

Within the UK Biobank, incident disease status and death information were determined through linkage with hospital in-patient data, cancer registry records, and death registry records. Our primary outcome focused on event-free survival time to the first moderate to severe CKD event. CKD events included stages 3-5 and end-stage renal disease (ESRD), identified by ICD-10 (10th revision of the International Statistical Classification of Diseases and Related Health Problems) codes N18.0, N18.3, N18.4, and N18.5, ascertained from hospital in-patient records in either the primary or the secondary position. The secondary outcomes in our study encompassed the individual components of CKD, specifically CKD stage 3, CKD stage 4, and CKD stage 5 as well as ESRD.

All participants were monitored from the date of recruitment (spanning from 2006 to 2010) to the occurrence of a CKD diagnosis, death, or the conclusion of the follow-up period (September 30, 2021, for England; July 31, 2021, for Scotland; and February 28, 2018, for Wales), whichever transpired first.

### Exposures

Serum Lp(a) levels were assessed using an immunoturbidimetric assay (Beckman Coulter AU5800; Randox Laboratories). In accordance with guidelines, Lp(a) was categorized into 2 groups: low (<75 nmol/L) and high (≥75 nmol/L) [4,9,23].

Serum creatinine (mmol/L) and urinary creatinine (mmol/L) were determined through enzymatic analyses (Beckman Coulter AU5800). The eGFR was computed using the serum creatinine–based Chronic Kidney Disease Epidemiology Collaboration (CKD-EPI) equation [24]. UACR was calculated as the ratio of urinary albumin (in mg/L) to urinary creatinine, with the former measured by an immunoturbidimetric assay (Beckman Coulter AU5400). As per recommendations, we classified both eGFR (low-normal, <90 mL/min/1.73 m^2^ and high-normal, ≥90 mL/min/1.73 m^2^) and UACR (low-normal, <10 mg/g and high-normal, ≥10 mg/g) into 2 groups [3].

### Other Independent Variables

Additional baseline independent variables considered comprised sociodemographic factors, lifestyle details, comorbidities, medication use, and laboratory samples. Sociodemographic factors included age (in years), sex (male or female), Townsend Deprivation Index (TDI), ethnicity (White, Mixed, Asian, Black, Chinese, and others), college degree or higher (yes or no), and residential area (urban or rural).

Lifestyle variables were BMI, smoking status (never, previous, or current smoker), alcohol drinking status (never, previous, or current drinker), regular vitamin supplement consumption (yes or no), mineral supplement use (yes or no), and coffee intake (yes or no). Comorbidities comprised a previous history of cancer, nonhypertensive CVD, depression, diabetes mellitus (DM), hypertension, high cholesterol, and use of drugs (antidiabetic drugs, antihypertensive drugs, or cholesterol-lowering drugs). Laboratory samples included high-density lipoprotein (HDL)-cholesterol (HDL-C), LDL-cholesterol (LDL-C), triglycerides (TGs), C-reactive protein (CRP), glycated hemoglobin (HbA_1c_), and urate. Table S1 in Multimedia Appendix 1 provides details on the aforementioned variables.

Data on sociodemographic factors and lifestyles were obtained through participant self-reports at baseline interviews. Information on baseline comorbidities and drug usage was gathered from participant self-reports, hospital in-patient records at baseline, and the relevant treatment/medication received. Laboratory samples, including blood and urine, were collected during participant recruitment.

### Statistical Analyses

Baseline characteristics of the included participants were presented as mean (SD) for continuous variables or frequency (percentage) for categorical variables. Chi-square and independent *t* tests (2-tailed) were performed to compare categorical and continuous variables, respectively, stratified by low and high Lp(a) groups.

Effect modification analyses were conducted to assess whether the impact of the high Lp(a) group on CKD risk varied within the strata of eGFR or UACR. We observed significant modifications by UACR (*P*=.02 for relative excess risk due to interaction), indicating that the association between Lp(a) and CKD risk was influenced by UACR levels, while no significant modifications were found by eGFR (*P*=.12 for relative excess risk due to interaction; refer to Tables S2 and S3 in Multimedia Appendix 1). When considering Lp(a), UACR, and eGFR as continuous variables, a significant interaction was observed only between Lp(a) and UACR (*P*=.03), while no significant interaction was found between Lp(a) and eGFR (*P*=.27) concerning CKD risk (refer to Table S4 in Multimedia Appendix 1). Subsequently, we delved into exploring the joint effect of Lp(a) and UACR on the risk of CKD through further analyses categorizing participants into 4 groups: low Lp(a) and low-normal UACR, low Lp(a) and high-normal UACR, high Lp(a) and low-normal UACR, and high Lp(a) and high-normal UACR.

Multivariable Cox proportional hazards models were used to explore the joint associations between Lp(a), UACR, and the risk of CKD, using the low Lp(a) and low-normal UACR group as the reference. The fully adjusted models included covariate adjustments for age, sex, BMI, TDI, college degree, ethnicity, area, smoking and drinking status, regular intake of coffee, vitamin and mineral supplements, personal medical history of cancer, CVD, depression, DM, hypertension, high cholesterol, drugs for DM, hypertension, high cholesterol, systolic blood pressure, HDL-C, LDL-C, TGs, HbA_1c_, CRP, urate, and eGFR. The variables included in the models were selected based on clinical expertise, prevailing research practices, and statistical knowledge [7-12]. The results were presented as hazard ratios (HRs) along with their corresponding 95% CIs. Additionally, a parsimonious model was used, adjusting only for age, sex, BMI, comorbidities, use of drugs, and eGFR, to evaluate the consistency of results with those from the fully adjusted models.

Subgroup analyses were performed to investigate the relationship between Lp(a), UACR, and CKD risk, stratified by sex (male vs female), age (<65 vs ≥65 years), medical history of DM (yes vs no), medical history of hypertension (yes vs no), and medical history of high cholesterol (yes vs no). In the subgroup analysis involving participants with DM, additional adjustment was made for their DM duration, considering the close association between DM duration and kidney function [25].

Several sensitivity analyses were conducted to assess the robustness of the main results. Initially, considering the potential correlation between LDL-C and Lp(a), we conducted the same analyses in multivariable models, excluding LDL-C to mitigate potential multicollinearity. Additionally, a sensitivity analysis was performed by further adjusting for moderate-to-vigorous physical activity and sleep patterns. Sleep pattern was defined based on a previous study using the UK Biobank cohort and incorporating 5 sleep behaviors: chronotype, duration, insomnia, snoring, and excessive daytime sleepiness [26]. Furthermore, we adjusted for family history of kidney diseases and the use of drugs for kidney diseases, including angiotensin-converting enzyme inhibitors and angiotensin receptor blockers, in another sensitivity analysis. Although comorbidities, including obesity, DM, and hypertension at baseline, were adjusted for in the models, it is possible that some participants without these comorbidities at baseline developed them during the 12.5-year follow-up. To address this, we conducted a sensitivity analysis by excluding participants who were free of these comorbidities at baseline but developed obesity, DM, or hypertension during the follow-up. This was done to minimize the dynamic impact of these comorbidities on the association between Lp(a), UACR, and CKD risk. We conducted a Fine-Gray competing risk analysis, treating all-cause mortality as a competing event for CKD [27]. To address reverse causation, we repeated the Cox regression analyses after excluding CKD events that occurred within the first year and the first 3 years of follow-up. Additionally, as another sensitivity analysis, we used multiple imputation techniques for missing data (seed=12345) to assess the robustness of the main findings. According to the guideline [28], participants with normal indicators (eGFR≥60 mL/min/1.73 m^2^ and UACR<30 mg/g) can be further diagnosed with CKD stage 1 or 2 if they exhibit other markers of kidney damage (eg, hematuria, electrolyte abnormalities, or structural abnormalities detected by imaging such as polycystic or dysplastic kidneys). Unfortunately, due to the unavailability of specific disease markers, we were unable to fully identify participants who should be diagnosed with CKD stage 1 or 2 at baseline. Consequently, we conducted another sensitivity analysis by including participants with a baseline eGFR≥90 mL/min/1.73 m^2^ in the analyses to exclude those with suspected CKD stage 1 or 2.

All tests were 2-sided with a significance level of .05. Statistical analyses were performed using SAS software version 9.4 (SAS Institute Inc.) and R software version 4.1.2 (R Foundation).

### Ethical Considerations

The UK Biobank study was approved by the North West Multicenter Research Ethics Committee (reference number: 16/NW/0274). All participants provided written consent before enrollment. The present analysis has received an exemption from the Research Ethics Committee of Guangdong Second Provincial General Hospital (reference number: 2022-KY-KZ-119-01) because it was a secondary analysis based on open data according to current regulations.

## Results

A total of 329,415 participants (mean age 56 years; n=175,117, 53.15% females) without prior CKD were included in the analyses. Among them, 258,388 (78.44%) had low Lp(a), while 71,027 (21.56%) had high Lp(a). Table 1 and Table S5 in Multimedia Appendix 1 provide descriptions and comparisons of baseline characteristics. Participants with high Lp(a) exhibited higher BMI, HDL-C, LDL-C, and HbA_1c_ levels, and were more likely to have a previous history of CVD and high cholesterol compared with the low Lp(a) group.

**Table 1:** Baseline characteristics of included participants.

Characteristics			Total (n=329,415)		Low Lp(a)^a,b^ (n=258,388)		High Lp(a) (n=71,027)		*P* value
Male sex, n (%)			154,298 (46.84)		120,890 (46.79)		33,408 (47.04)		.20
Age (years), mean (SD)			56.3 (8.09)		56.3 (8.09)		56.1 (8.13)		<.001
BMI (kg/m^2^), mean (SD)			27.3 (4.66)		27.3 (4.65)		27.4 (4.71)		<.001
**Townsend Deprivation Index** **, mean (SD)**			–1.35 (3.07)		–1.37 (3.05)		–1.29 (3.12)		<.001
	Median (Q1 to Q3)	–2.18 (–3.67 to 0.46)		–2.19 (–3.67 to 0.42)		–2.14 (–3.65 to 0.55)			
College degree or higher, n (%)			108,671 (32.99)		85,368 (33.04)		23,303 (32.81)		.25
Urban area, n (%)			280,042 (85.01)		219,554 (84.97)		60,488 (85.16)		.08
**Ethnicity, n (%)**									<.001
	White	309,923 (94.08)		244,101 (94.47)		65,822 (92.67)			
	Mixed	2016 (0.61)		1574 (0.61)		442 (0.62)			
	Asian	6536 (1.98)		5212 (2.02)		1324 (1.86)			
	Black	5315 (1.61)		3038 (1.18)		2277 (3.21)			
	Chinese	1112 (0.34)		985 (0.38)		127 (0.18)			
	Others	3027 (0.92)		2326 (0.90)		701 (0.99)			
**Moderate-to-vigorous physical activity** **(** **metabolic equivalent of task** **, minutes/week), n (%)**									.48
	0	35,993 (10.93)		28,260 (10.94)		7733 (10.89)			
	1-599	68,698 (20.85)		53,928 (20.87)		14,770 (20.79)			
	600-1199	45,633 (13.85)		35,869 (13.88)		9764 (13.75)			
	≥1200	117,130 (35.56)		91,704 (35.49)		25,426 (35.80)			
**Smoking status, n (%)**									.06
	Never	181,057 (54.96)		141,843 (54.90)		39,214 (55.21)			
	Previous	112,575 (34.17)		88,571 (34.28)		24,004 (33.80)			
	Current	34,217 (10.39)		26,773 (10.36)		7444 (10.48)			
**Drinking status, n (%)**									.02
	Never	14,099 (4.28)		10,924 (4.23)		3175 (4.47)			
	Previous	11,196 (3.40)		8776 (3.40)		2420 (3.41)			
	Current	303,380 (92.10)		238,125 (92.16)		65,255 (91.87)			
Coffee intake, n (%)			256,104 (77.75)		201,200 (77.87)		54,904 (77.30)		.001
Vitamin supplement, n (%)			103,367 (31.38)		80,797 (31.27)		22,570 (31.78)		.009
Mineral supplement, n (%)			140,300 (42.59)		110,173 (42.64)		30,127 (42.42)		.28
**Sleep pattern, n (%)**									
	Poor	6302 (1.91)		4926 (1.91)		1376 (1.94)		.84	
	Intermediate	106,559 (32.35)		83,610 (32.36)		22,949 (32.31)			
	Healthy	159,283 (48.35)		124,911 (48.34)		34,372 (48.39)			
Cancer, n (%)			36,744 (11.15)		29,065 (11.25)		7679 (10.81)		.001
Cardiovascular disease, n (%)			41,465 (12.59)		32,133 (12.44)		9535 (13.42)		<.001
Depression, n (%)			15,254 (4.63)		4282 (1.66)		19,536 (27.51)		.32
Diabetes mellitus, n (%)			25,129 (7.63)		19,721 (7.63)		5408 (7.61)		.97
Course of diabetes, mean (SD)			9.69 (13.2)		9.74 (13.2)		9.49 (13.0)		.37
Hypertension, n (%)			87,697 (26.62)		68,541 (26.53)		19,156 (26.97)		.06
High cholesterol, n (%)			8562 (2.60)		6417 (2.48)		2145 (3.02)		<.001
Family history of kidney disorders, n (%)			7 (0.00)		5 (0.00)		2 (0.00)		>.99
Use of antidiabetic drugs, n (%)			2789 (0.85)		2217 (0.86)		572 (0.81)		.28
Use of antihypertensive drugs, n (%)			61,759 (18.75)		48,152 (18.64)		13,607 (19.16)		.01
Use of cholesterol-lowing drugs, n (%)			50,655 (15.38)		38,970 (15.08)		11,685 (16.45)		<.001
Use of angiotensin-converting enzyme inhibitor/angiotensin receptor blocker, n (%)			46 (0.01)		37 (0.01)		9 (0.01)		.88
High-density lipoprotein cholesterol (mmol/L), mean (SD)			1.44 (0.38)		1.44 (0.38)		1.45 (0.38)		<.001
Low-density lipoprotein cholesterol (mmol/L), mean (SD)			3.57 (0.86)		3.55 (0.85)		3.63 (0.86)		<.001
Triglycerides (mmol/L), mean (SD)			1.74 (1.01)		1.75 (1.02)		1.68 (0.98)		<.001
C-reactive protein (mg/L), mean (SD)			2.51 (4.18)		2.51 (4.19)		2.50 (4.14)		.57
Direct glucose (mmol/L), mean (SD)			5.08 (1.12)		5.08 (1.12)		5.08 (1.13)		.53
Glycated hemoglobin (mmol/mol), mean (SD)			35.80 (6.15)		35.8 (6.16)		35.9 (6.13)		.02
Urate (μmol/L), mean (SD)			308 (77.90)		308 (78.01)		308 (77.79)		.77
Estimated glomerular filtration rate (mL/min/1.73 m^2^), mean (SD)			91.7 (12.01)		91.6 (12.08)		91.7 (12.20)		.35
Urine albumin-creatinine ratio (mg/g), mean (SD)			10.90 (6.30)		10.9 (6.31)		10.8 (6.26)		<.001

^a^Lp(a): lipoprotein a.

^b^Baseline Lp(a) was categorized into low (< 75 nmol/L) and high (≥ 75 nmol/L) groups.

There were 6003 incident CKD events recorded, with a median follow-up of 12.5 years and a total of 4,011,201 person-years. Table 2 (also see Multimedia Appendix 2) presents the results for the independent associations between Lp(a), UACR, and CKD risk. The high Lp(a) group showed a nonsignificant association with an elevated CKD risk (HR 1.05, 95% CI 0.98-1.13; *P*=.16) compared with the low Lp(a) group. By contrast, the high-normal UACR group was significantly associated with a 20% increased risk of CKD compared with the low-normal UACR group (HR 1.20, 95% CI 1.13-1.27; *P*<.001). Similar results were observed when treating Lp(a) and UACR as continuous variables (see Table S4 in Multimedia Appendix 1).

**Table 2:** Associations between Lp(a)^a^, UACR^b^, and risks of chronic kidney disease.

Variables			Number of cases/total participants		HR^c^ (95% CI); *P* value^d^
**Independent associations**					
**Lp(a)**					
	Low group	4642/258,388		Reference	
	High group	1361/71,027		1.05 (0.98-1.13); .16	
**UACR**					
	Low-normal group	3247/182,740		Reference	
	High-normal group	2756/146,675		1.20 (1.13-1.27); <.001	
**Joint associations between baseline Lp(a)^e^ and UACR**					
	Low Lp(a) and low-normal UACR	2527/142,923		Reference	
	High Lp(a) and low-normal UACR	720/39,817		0.98 (0.90-1.08); .74	
	Low Lp(a) and high-normal UACR	2115/15,465		1.16 (1.08-1.24); <.001	
	High Lp(a) and high-normal UACR	641/31,210		1.32 (1.19-1.46); <.001	

^a^Lp(a): lipoprotein a.

^b^UACR: urine albumin-creatinine ratio.

^c^HR: hazard ratio.

^d^Fully adjusted model adjusted for age, sex, body mass index, Townsend Deprivation Index, college degree, ethnicity, area, smoking and drinking status, regular intake of coffee, vitamin and mineral supplement, personal medical history of cancer, cardiovascular disease, depression, diabetes, hypertension, high cholesterol, drugs for diabetes, hypertension, high cholesterol, systolic blood pressure, high-density lipoprotein cholesterol, low-density lipoprotein cholesterol, triglycerides, glycated hemoglobin (HbA_1c_), C-reactive protein, urate, and estimated glomerular filtration rate.

^e^Baseline Lp(a) was categorized into low (<75 nmol/L) and high (≥75 nmol/L) groups. Baseline UACR within the normal range was classified into low-normal (0-9.9 mg/g) and high-normal (10-29.9 mg/g) groups.

The joint associations between Lp(a) and UACR, as demonstrated through the 4 groups generated from their cross-categorization, with low Lp(a) and low-normal UACR as the reference group, are shown in Table 2. Among participants with low-normal UACR, a nonsignificant association was observed between high Lp(a) and CKD risk (HR 0.98, 95% CI 0.90-1.08; *P*=.74). When compared with the low Lp(a) and low-normal UACR group, both the low Lp(a) and high-normal UACR and high Lp(a) and high-normal UACR groups were significantly associated with increased risks of CKD, with HRs of 1.16 (95% CI 1.08-1.24; *P*<.001) and 1.32 (95% CI 1.19-1.46; *P*<.001), respectively. Table S2 in Multimedia Appendix 1 shows that a significant association between Lp(a) and increased CKD risk was only observed in the high-normal UACR group (HR 1.14, 95% CI 1.03-1.26; *P*=.01), but not in the low-normal UACR group (HR 0.98, 95% CI 0.90-1.08; *P*=.74). Similar results from the parsimonious model were found to support our main findings (Table S6 in Multimedia Appendix 1).

**Figure 1:**
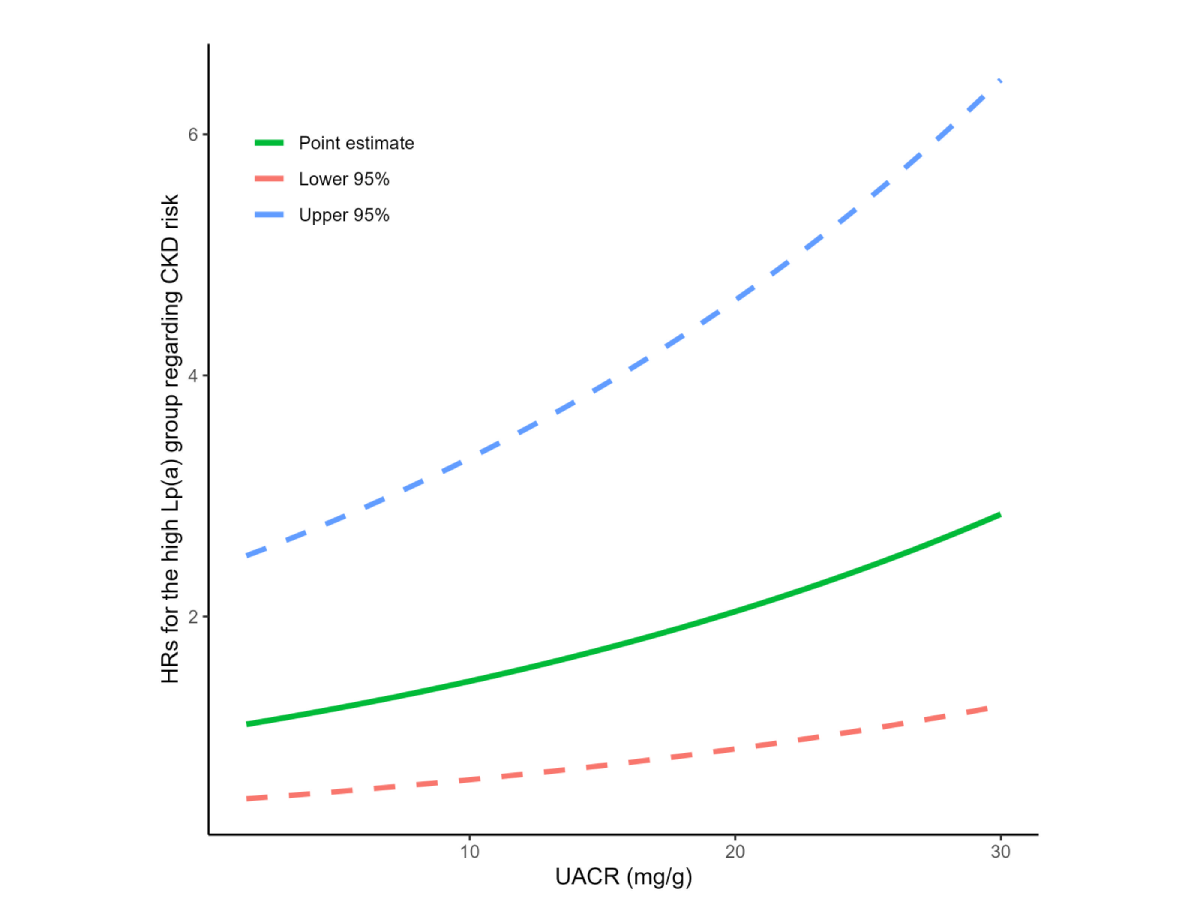
HRs for the high Lp(a) group regarding the risk of chronic kidney disease at different levels of UACR. CKD: chronic kidney disease; HR: hazard ratio; lower 95%: lower limit of 95% CI; Lp(a): lipoprotein(a); UACR: urine albumin-to-creatinine ratio; upper 95%: upper limit of 95% CI.

When treating UACR as a continuous variable, increased HRs regarding the relationship between the high Lp(a) group and CKD risk were observed as UACR elevated (Figure 1), indicating that an increased UACR modifies the propensity of high Lp(a) toward CKD risk when compared with low Lp(a).

**Figure 2:**
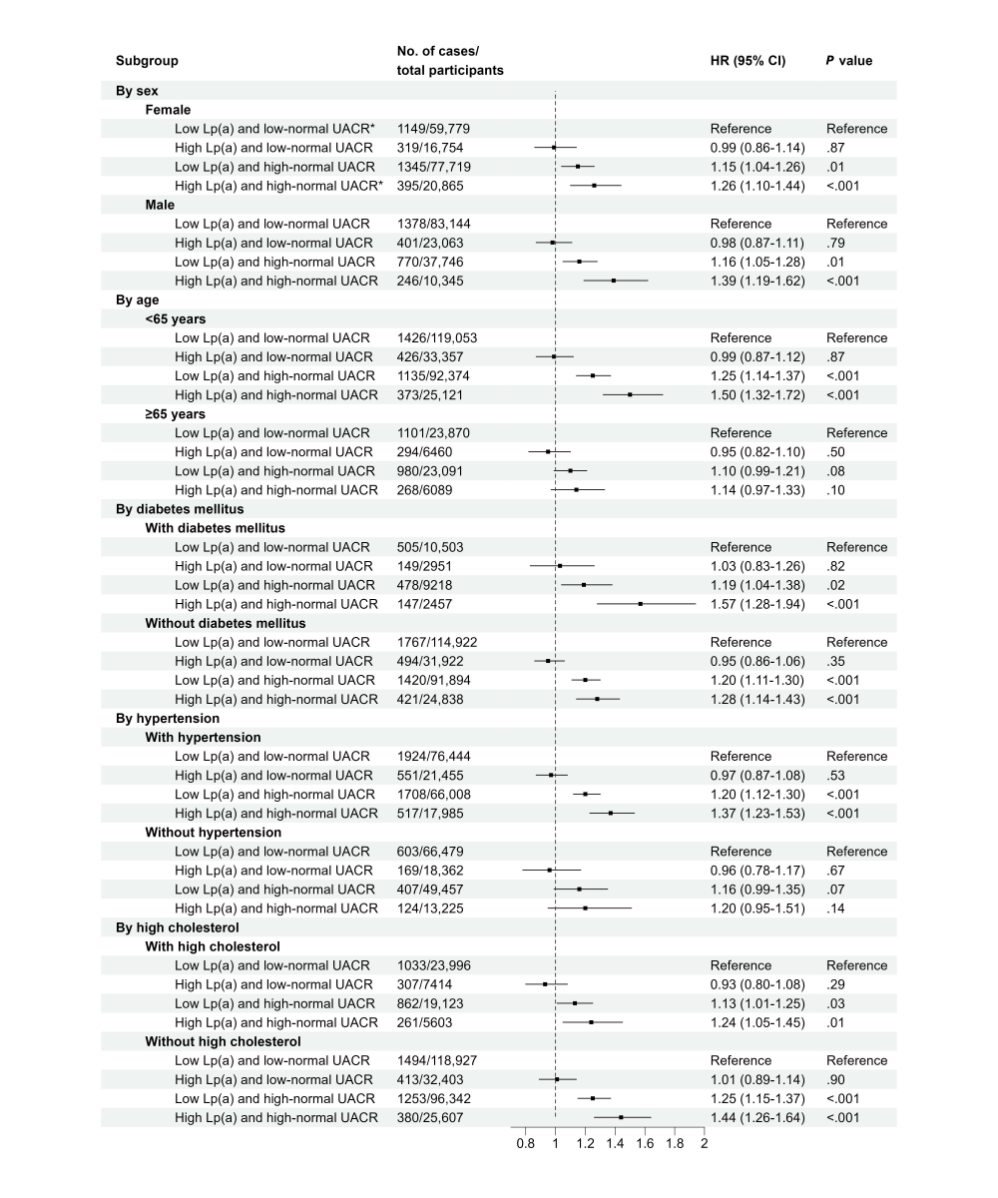
Stratified analyses of joint associations between baseline Lp(a) and UACR regarding the risk of chronic kidney disease. *Baseline Lp(a) was categorized into low (<75 nmol/L) and high (≥75 nmol/L) groups. Baseline UACR within the normal range was classified into low-normal (0-9.9 mg/g) and high-normal (10-29.9 mg/g) groups. HR: hazard ratio; Lp(a): lipoprotein(a); UACR: urine albumin-to-creatinine ratio.

**Figure 3:**
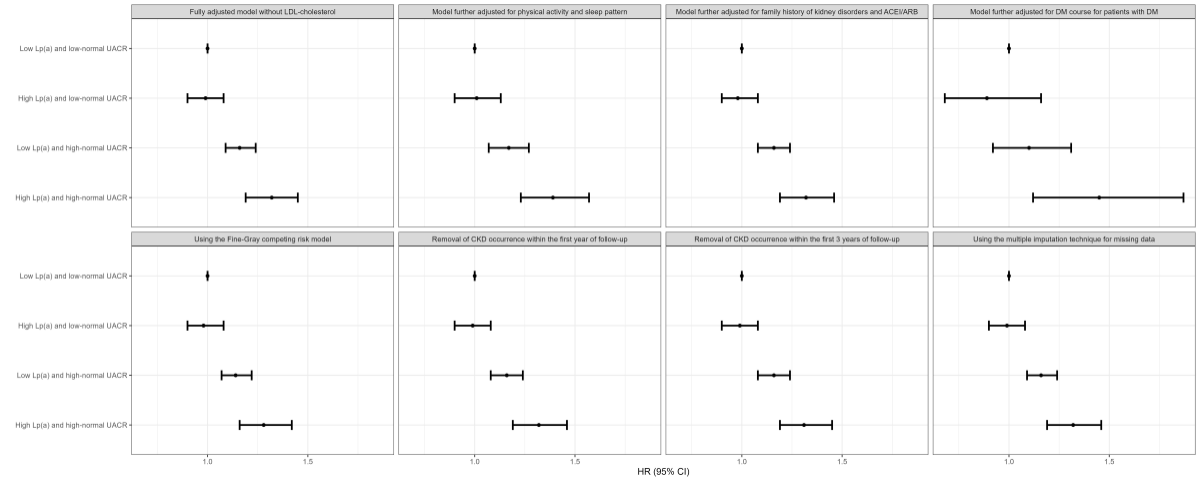
Further analyses of joint associations between baseline Lp(a) and UACR regarding the risk of CKD. *Baseline Lp(a) was categorized into low (<75 nmol/L) and high (≥75 nmol/L) groups. Baseline UACR within the normal range was classified into low-normal (0-9.9 mg/g) and high-normal (10-29.9 mg/g) groups. ACEI: angiotensin-converting enzyme inhibitor; ARB: angiotensin receptor blocker; CKD: chronic kidney disease; DM: diabetes mellitus; Lp(a): lipoprotein(a); UACR: urine albumin-to-creatinine ratio.

Figure 2 shows the joint associations between Lp(a) and UACR with the risk of CKD in different subgroups. Results similar to the main findings were observed within different strata of sex, previous history of DM, and high cholesterol. Among participants aged ≥65 years or without a previous history of hypertension, both the low Lp(a) and high-normal UACR (*P*=.08 for participants aged ≥65 years and *P*=.07 for participants without hypertension) and high Lp(a) and high-normal UACR (*P*=.10 for participants aged ≥65 years and *P*=.14 for participants without hypertension) groups were nonsignificantly associated with increased risks of CKD. Sensitivity analyses yielded largely similar results to the main findings (Figure 3 and Tables S7 and S8 in Multimedia Appendix 1).

Table S9 in Multimedia Appendix 1 shows results for secondary outcomes (5615 with CKD stage 3, 393 with CKD stage 4, and 252 with CKD stage 5 and ESRD), with similar findings to the primary outcome in general.

## Discussion

### Principal Findings

In this study based on data from a prospective cohort, our principal findings are as follows: (1) high Lp(a) was nonsignificantly associated with increased CKD risk among all participants; (2) there was a significant interaction between Lp(a) and UACR but not between Lp(a) and eGFR; (3) when taking low Lp(a) and low-normal UACR as the reference, the high-normal UACR groups with any Lp(a) level were significantly associated with an increased risk of CKD, with no significant risks observed in the high Lp(a) and low-normal UACR group; (4) among those with high-normal UACR, high Lp(a) was associated with a significant increase in CKD risk; and (5) when treating UACR as a continuous variable, increased HRs regarding the relationship between Lp(a) and CKD risk were observed as UACR elevated.

Our study shows that the association between Lp(a) and CKD risk was nonsignificant among the general population without previous CKD in both categorical and continuous forms. Indeed, the relationship between Lp(a) and CKD risk has been explored, with inconsistent findings reported. One Chinese cohort study, including 6257 adults, showed that elevated Lp(a) was significantly associated with an increased risk of reduced renal function [10]. Nevertheless, the DiaGene study, including participants with type 2 DM, found that neither the high baseline Lp(a) group (also defined as ≥75 nmol/L) nor 2 related Lp(a) single-nucleotide polymorphisms were significantly related to the risk of incident nephropathy [9]. Inconsistent findings have also been reported in several Mendelian randomization studies. For instance, the study by Zheng et al [14] supported the causal role of Lp(a) in CKD development, while another study did not detect a significant association between Lp(a) and the risk of nephropathy [13]. These discrepant findings may partly be due to the heterogeneity of the target populations, outcome definitions, and statistical analyses. Although some previous studies adjusted for renal function indicators as covariates in their models [7-10,12], the relationship between Lp(a) and CKD risk was indeed assessed based on the average level of renal function in their populations. Thus, previous studies found different associations between Lp(a) and the risk of CKD, probably depending on the various average levels of renal function at baseline.

By contrast, we found a significant interaction between Lp(a) and UACR, and more specifically, a synergistic effect between Lp(a) and UACR on the risk of CKD. Interestingly, when the UACR value exceeded 10 mg/g approximately, the relationship between high Lp(a) and CKD risk became significant (Figure 1 and Table S2 in Multimedia Appendix 1), supporting the modification of UACR to the association between Lp(a) and CKD risk. Of note, Lp(a) together with other lipid and lipoprotein concentrations such as LDL particles had been reported to associate with UACR elsewhere, even among participants with a normal UACR range [29-31]. Therefore, it was plausible that UACR could act downstream on the pathway from Lp(a) to CKD. To address this concern, we performed a post hoc mediation analysis using a generalized linear model with 100 bootstrapping times [32,33]. The total effect of Lp(a) on CKD risk was 0.199 (95% CI 0.007-0.399), with the average direct effect of 0.195 (95% CI 0.008-0.373) and the average causal mediation effect through UACR of 0.004 (95% CI –0.005 to 0.014), indicating a nonsignificant mediation effect. This could further support the joint relationship between Lp(a) and UACR regarding CKD risk from a public health perspective. Therefore, our results may highlight the importance of UACR when clarifying Lp(a) in relation to the risk of CKD in population studies, even if the participants were free of a CKD diagnosis and their renal function indicators were within the normal range.

Nevertheless, why the relationship between Lp(a) and CKD risk depended on UACR remained largely unknown. A possible mechanism might be that elevated Lp(a) could be linked to increased synthesis of proteins in the liver due to proteinuria [34], where high UACR could be closely associated with proteinuria. There appeared to be an interaction between Lp(a) and hypertension regarding CVD outcomes [35], where hypertension had been reported to be significantly associated with elevated normal UACR [36]. Nevertheless, no significant subgroup effect by the existence of hypertension was found in our subgroup analysis (Figure 2). Of note, evidence from basic science might help explain the interaction. Lp(a) could enhance the expression of adhesion molecules in endothelial cells and aggravate normal endothelial function [37,38]. UACR serves as a common marker of endothelial and kidney function [39], and is reported to be an earlier and greater marker for some kidney outcomes than eGFR [40-42]; therefore, the detrimental effect of Lp(a) on the progression of CKD may be only significantly observed in those with high-normal UACR who had impaired renal function of clearance and self-recovery. However, the underlying mechanisms of the effects of Lp(a) and UACR on CKD risk have not been extensively elucidated, necessitating additional population studies for further exploration and clarification.

In participants aged over 65 years, high-normal UACR groups showed a nonsignificant association with CKD risk (Figure 2), consistent with findings from a large collaborative meta-analysis [19]. Moreover, the relationship between Lp(a) and CKD risk in patients with DM has been extensively investigated, yielding inconsistent results [11]. In our study, among participants with a history of DM, a significant association between Lp(a) and CKD risk was observed in the high-normal UACR group, but not in the low-normal UACR group (Figure 2). Hence, once more, examining the interaction between Lp(a) and UACR concerning CKD risk in participants with DM could contribute to understanding the prior inconclusive results in public health studies, where diverse baseline renal function existed across populations. Nonetheless, it is crucial to approach the results of our subgroup analyses with caution, considering their exploratory nature and their role in generating hypotheses.

CKD has emerged as a significant public health concern, characterized by a widespread prevalence and a substantial global disease burden [43]. The imperative now is to urgently improve early detection and preventive measures for CKD, given the considerable costs associated with therapy and the elevated mortality rates linked to advanced CKD stages [43]. Providing clarity on the role of Lp(a) could contribute significantly to public initiatives focused on preventing and managing the risks associated with CKD. Nevertheless, it is noteworthy that Lp(a) remains unaddressed in existing public health policies or guidelines for CKD [3,15]. It is widely acknowledged that serum Lp(a) levels are predominantly genetically determined, showing no significant associations with environmental or lifestyle factors [44,45]. While certain medications, such as muvalaplin, have shown effectiveness in lowering Lp(a) levels, their safety, tolerability, and cost-effectiveness still lack comprehensive clarity [46-48]. Addressing these uncertainties calls for more extensive and prolonged clinical trials in the future. Hence, there is a particular need for public health interventions, focusing on improving the management of both Lp(a) and UACR, as well as enhancing the accessibility of kidney health therapies. Furthermore, there is a necessity for quantitative assessment to determine the CKD risk attributed to Lp(a) in diverse populations. Such evaluations could strengthen the case for considering Lp(a) in the screening and management protocols for CKD. Similarly, should our findings be externally validated, the incorporation of Lp(a) into strategies could aid in targeting populations at a heightened risk of CKD, thereby bolstering efforts in CKD prevention.

### Strengths

Our study carries several strengths. First, we leveraged data from a nationwide cohort, providing a substantial amount of information for our analyses. The application of rigorous methodology underpins the validity and robustness of our results. Notably, this study represents the first attempt to explore the connection between Lp(a), UACR, and the risk of CKD. By doing so, we aimed to elucidate the intricate relationship between Lp(a) and CKD risk within the general population, offering valuable insights for CKD risk assessment and prevention. This perspective, grounded in public health, sheds light on the role of Lp(a) and a renal function indicator in shaping strategies for CKD prevention.

### Limitations

Several limitations should be acknowledged. First, owing to the observational design of our study, it is crucial to recognize that potential bias or confounding effects could not be entirely mitigated, and establishing a causal relationship between Lp(a) and CKD risk was beyond the scope of our investigation. Second, because both Lp(a) and UACR data were gathered at baseline, our analyses could not explore whether Lp(a) serves as a marker for UACR in the context of CKD risk. In addition, our study lacked the capacity to analyze changes in Lp(a) and UACR concerning the risk of CKD. Furthermore, Lp(a) measurements were conducted using a widely available immunoassay method, potentially introducing measurement errors due to the heterogeneity of isoform size when compared with the gold-standard method used by the Northwest Lipid Metabolism Diabetes Research Laboratory [49]. Similarly, there might be measurement errors for UACR, calculated as the ratio between urinary albumin and creatinine. The assays used for urinary albumin and creatinine involved an immunoturbidimetric method and enzymatic analysis, respectively [50]. As CKD outcomes were determined using records from hospital in-patient data, cancer registries, and death registries, there is a possibility that CKD events were inadequately estimated due to underdiagnosis, misdiagnosis, or incorrect coding, the extent of which remains unknown. In addition, it is important to note that the UK Biobank, from which our participants were drawn, had over 90% (309,923/329,415, 94.08%) of individuals identifying as White. As a result, the generalizability of our study results to other racial groups may be compromised.

### Conclusions

A notable interaction was identified between Lp(a) and UACR concerning the risk of CKD. This implies that Lp(a) may serve as a risk factor for CKD even when considering the influence of UACR. Our results offer valuable insights into the assessment and prevention of CKD, emphasizing the combined role of Lp(a) and UACR from a public health perspective within the general population. This perspective can contribute to enhancing public awareness regarding the management of Lp(a) for the prevention of CKD.

## Appendix

Multimedia Appendix 1 Supplementary materials for Relationship Between Lipoprotein(a), Renal Function Indicators, and Chronic Kidney Disease: Evidence From a Large Prospective Cohort Study.

Multimedia Appendix 2 The Strengthening the Reporting of Observational studies in Epidemiology (STROBE) checklist.

## Abbreviations

CKD: chronic kidney disease
CKD-EPI: Chronic Kidney Disease Epidemiology Collaboration
CRP: C-reactive protein
CVD: cardiovascular disease
DM: diabetes mellitus
eGFR: estimated glomerular filtration rate
ESRD: end-stage renal disease
HbA1c: glycated hemoglobin
HDL: high-density lipoprotein
HR: hazard ratio
ICD-10: 10th revision of the International Statistical Classification of Diseases and Related Health Problems
LDL: low-density lipoprotein
Lp(a): lipoprotein(a)
TDI: Townsend Deprivation Index
TG: triglyceride
UACR: urine albumin-to-creatinine ratio

## Data Availability Statement

The data can be obtained by sending an application to the UK Biobank [22].
