# Supplementary material for: Relationship Between Lipoprotein(a), Renal Function Indicators, and Chronic Kidney Disease: Evidence From a Large Prospective Cohort Study
Source: JMIR Public Health Surveill. 2024 Jan 31;10:e50415. doi: 10.2196/50415 (PMC10867749; doi:10.2196/50415)

Contents

STable 1. Ascertainment of Baseline Characteristics 2
STable 2. Effect modification of Lp(a) on risk of chronic kidney disease by UACR* 3
STable 3. Effect modification of Lp(a) on risk of chronic kidney disease by eGFR* 4
STable 4. Associations between Lp(a), UACR, eGFR and risk of chronic kidney disease when taking Lp(a), UACR, eGFR as continuous variables* 5
STable 5. Baseline characteristics of participants by UACR and eGFR groups 6
STable 6. Associations between Lp(a), UACR and risks of chronic kidney disease using a parsimonious model 8
STable 7. Associations between Lp(a), UACR and risks of chronic kidney disease in participants with baseline eGFR over 90 mL/min/1.73 m^2 9
STable 8. Associations between Lp(a), UACR and risks of chronic kidney disease after removal of several disease occurrences during follow up of the study 10
STable 9. Associations between Lp(a), UACR and risks of secondary outcomes 11
SFigure 1. Flowchart for the study design 12

STable 1. Ascertainment of Baseline Characteristics

Characteristics	Category	Source	Code
**Hypertension**	Markers	Baseline: DBP ≥ 90	-
		Baseline: SBP ≥ 140	-
	Disease	HES: ICD10	I10-I15
		HES: ICD9	401-405
		Baseline: Vascular/heart (6150)	4
		Baseline: non-cancer illness code (20002)	1065, 1072
	Medication	Baseline: Medications for diseases (6153)	2
		Baseline: Medications for diseases (6177)	2
		Baseline: Treatment/Medication (20003)	1140888578
**Diabetes mellitus**	Markers	Baseline: fast glucose ≥ 7 mmol/L	-
		Baseline: HbA1c ≥ 6.5 %	-
	Disease	HES: ICD10	E10-E14
		HES: ICD9	250
		Baseline: diabetes (2443)	1
		Baseline: non-cancer illness code (20002)	1220-1223,1521
	Medication	Baseline: Medications for diseases (6153)	3
		Baseline: Medications for diseases (6177)	3
		Baseline: Treatment/Medication (20003)	1140883066
**High cholesterol**	Disease	HES: ICD10	E780
		HES: ICD9	250
		Baseline: non-cancer illness code (20002)	1473
	Medication	Baseline: Medications for diseases (6153)	1
		Baseline: Medications for diseases (6177)	1
		Baseline: Treatment/Medication (20003)	1140861942<br>1140865576<br>1141157416
**Non-hypertensive CVD**	Disease	HES: ICD10	I20-I52,<br>I70-I89,<br>I95-I99
		HES: ICD9	390-400,<br>406-459
		Baseline: Vascular/heart (6150)	1-3
		Baseline: non-cancer illness code (20002)	1066-1068,<br>1074-1094,<br>1425-1495,<br>1583-1593
**Depression**	disease	HES: ICD10	F32-F33
		Baseline: depression status (20126)	>2
		Baseline: mental health problem (20544)	11
		Baseline: non-cancer illness code (20002)	1286,1531

Abbreviations: HES, Hospital Episodes Statistics; ICD, International Classification of Disease; DBP, diastolic blood pressure; SBP, systolic blood pressure; CVD, cardiovascular disease

STable 2. Effect modification of Lp(a) on risk of chronic kidney disease by UACR*

	**Low Lp(a)**		**High Lp(a)**		**Effect of high Lp(a) within strata of UACR**	**Additive measures[1]**			**Multiplicative measure[2]**
	**No. of cases / total participants**	**HR (95% CI), p-value**	**No. of cases / total participants**	**HR (95% CI), p-value**	**HR (95% CI), p-value**	**RERI (95% CI), p-value**	**AP (95% CI), p-value**	**S (95% CI), p-value**	**The ratio of HR (95% CI), p-value**
**Low-normal UACR**	2,527/14,2923	Ref	720/39,817	0.98 (0.90, 1.08), p = 0.742	0.98 (0.9, 1.08), p = 0.742	0.17 (0.02, 0.33), p = 0.015	0.13 (0.02, 0.24), p = 0.010	2.20 (0.88, 5.49), p < 0.001	1.15 (1.01, 1.32), p = 0.041
**High-normal UACR**	2,115/115,465	1.16 (1.08, 1.24), p < 0.001	641/31,210	1.32 (1.19, 1.46), p < 0.001	1.14 (1.03, 1.26), p = 0.013				
**Effect of high-normal UACR within strata of Lp(a)**		1.16 (1.08, 1.24), p < 0.001		1.34 (1.18, 1.51), p < 0.001					

Abbreviations: UACR, urine albumin-creatinine ratio; Lp(a), lipoprotein a; HR, hazard ratio; CI, confidence interval; RERI, relative excess risk due to interaction; AP, attributable proportion due to interaction; S, the synergy index.

*Baseline Lp(a) was categorized into low (< 75 nmol/L) and high (≥ 75 nmol/L) groups. Baseline UACR within the normal range was classified into low-normal (0 - 9.9 mg/g) and high-normal (10 - 29.9 mg/g) groups

*Models adjusted for sex, age, body mass index, Townsend Deprivation Index, college degree, ethnicity, area, smoking and drinking status, regular intake of coffee, vitamin and mineral supplement, personal medical history of cancer, CVD, depression, diabetes, hypertension, high cholesterol, drugs for diabetes, hypertension, high cholesterol, SBP, HDL cholesterol, LDL cholesterol, TG, HbA1c, CRP, urate, eGFR

[1]Significant interactions between high Lp(a) and UACR were detected on an additive scale using all three measures: RERI, AP, S

[2]Significant interaction between high Lp(a) and UACR was observed on a multiplicative scale

STable 3. Effect modification of Lp(a) on risk of chronic kidney disease by eGFR*

	**Low Lp(a)**		**High Lp(a)**		**Effect of high Lp(a) within strata of eGFR**	**Additive measures[1]**			**Multiplicative measure[2]**
	**No. of cases / total participants**	**HR (95% CI), p-value**	**No. of cases / total participants**	**HR (95% CI), p-value**	**HR (95% CI), p-value**	**RERI (95% CI), p-value**	**AP (95% CI), p-value**	**S (95% CI), p-value**	**The ratio of HR (95% CI), p-value**
**High-normal eGFR**	740/155,074	Ref	204/42,526	1.06 (0.89, 1.26), p = 0.544	1.06 (0.89, 1.26), p = 0.544	0.27 (-0.18, 0.73), p = 0.120	0.05 (-0.03, 0.13), p = 0.114	1.06 (0.96, 1.17), p = 0.060	1.01 (0.83, 1.22), p = 0.955
**Low-normal eGFR**	3,902/103,314	5.38 (4.90, 5.91), p < 0.001	1,157/28,501	5.71 (5.12, 6.37), p = 3.174	1.06 (0.98, 1.14), p = 0.121				
**Effect of low-normal eGFR within strata of Lp(a)**		5.38 (4.90, 5.91), p < 0.001		5.41 (4.57, 6.41), p < 0.001					

Abbreviations: eGFR, estimated glomerular filtration rate; Lp(a), lipoprotein a; HR, hazard ratio; CI, confidence interval; RERI, relative excess risk due to interaction; AP, attributable proportion due to interaction; S, the synergy index.

* Baseline Lp(a) was categorized into low (< 75 nmol/L) and high (≥ 75 nmol/L) groups. Baseline eGFR within the normal range was classified into low-normal (< 90 mL/min/1.73 m^2) and high-normal (≥ 90 mL/min/1.73 m^2) groups.

*Models adjusted for sex, age, body mass index, Townsend Deprivation Index, college degree, ethnicity, area, smoking and drinking status, regular intake of coffee, vitamin and mineral supplement, personal medical history of cancer, CVD, depression, diabetes, hypertension, high cholesterol, drugs for diabetes, hypertension, high cholesterol, SBP, HDL cholesterol, LDL cholesterol, TG, HbA1c, CRP, urate, UACR.

[1]Non-significant interactions between high Lp(a) and eGFR were detected on an additive scale using all three measures: RERI, AP, S

[2]Non-significant interaction between high Lp(a) and eGFR was observed on a multiplicative scale

STable 4. Associations between Lp(a), UACR, eGFR and risk of chronic kidney disease when taking Lp(a), UACR, eGFR as continuous variables*

**Variables**	**No. of cases / total participants**	**HR (95% CI), p-value**
Independent, Per SD increase		
Lp(a)	6,003 / 329,415	1.02 (0.99, 1.05), p = 0.163
UACR	6,003 / 329,415	1.14 (1.10, 1.17), p < 0.001
eGFR	6,003 / 329,415	0.35 (0.34, 0.37), p < 0.001
Interaction terms		
Lp(a)* UACR	6,003 / 329,415	1.04 (1.00, 1.08), p = 0.029
Lp(a)* eGFR	6,003 / 329,415	1.02 (0.98, 1.06), p = 0.269

Abbreviations: UACR, urine albumin-creatinine ratio; Lp(a), lipoprotein a; HR, hazard ratio; CI, confidence interval.
*Fully adjusted model adjusted for age, sex, body mass index, Townsend Deprivation Index, college degree, ethnicity, area, smoking and drinking status, regular intake of coffee, vitamin and mineral supplement, personal medical history of cancer, CVD, depression, diabetes, hypertension, high cholesterol, drugs for diabetes, hypertension, high cholesterol, SBP, HDL cholesterol, LDL cholesterol, TG, HbA1c, CRP, urate, eGFR.

STable 5. Baseline characteristics of participants by UACR and eGFR groups

Characteristics	UACR			eGFR		
	Low-normal group (n = 182,740)	High-normal group (n = 146,675)	P-value	Low-normal group (n = 131,815)	High-normal group (n = 197,600)	P-value
**Male Sex**	106207 (58.1)	48091 (32.8)	<0.001	62244 (47.2)	92054 (46.6)	<0.001
**Age (years)**	55.7 (8.19)	57.0 (7.91)	<0.001	59.4 (7.23)	54.2 (7.96)	<0.001
**BMI (kg/m2)**	27.9 (4.65)	26.7 (4.59)	<0.001	27.7 (4.47)	27.1 (4.77)	<0.001
**TDI**	-1.27 (3.12)	-1.45 (3.00)	<0.001	-1.55 (2.96)	-1.22 (3.13)	<0.001
Median (Q1,Q3)	**-2.11 (-3.63,0.59)**	**-2.25 (-3.70,0.27)**	**<0.001**	**-2.35 (-3.74,0.083**	**-2.05 (-3.61,0.69**	**<0.001**
**College degree or higher**	60032 (32.9)	48639 (33.2)	<0.001	39940 (30.3)	68731 (34.8)	<0.001
**Urban Area**	155867 (85.3)	124175 (84.7)	<0.001	110993 (84.2)	169049 (85.6)	<0.001
**Ethnicity**						
White	170942 (93.5)	138981 (94.8)	<0.001	126087 (95.7)	183836 (93.0)	<0.001
Mixed	1235 (0.7)	781 (0.5)		677 (0.5)	1339 (0.7)	
Asian	3497 (1.9)	3039 (2.1)		1884 (1.4)	4652 (2.4)	
Black	3877 (2.1)	1438 (1.0)		1431 (1.1)	3884 (2.0)	
Chinese	549 (0.3)	563 (0.4)		234 (0.2)	878 (0.4)	
Others	1787 (1.0)	1240 (0.8)		908 (0.7)	2119 (1.1)	
**MVPA (MET min/week)**						
0	20915 (11.4)	15078 (10.3)	<0.001	13926 (10.6)	22067 (11.2)	<0.001
1-599	38811 (21.2)	29887 (20.4)		26767 (20.3)	41931 (21.2)	
600-1199	24928 (13.6)	20705 (14.1)		18020 (13.7)	27613 (14.0)	
≥ 1200	65117 (35.6)	52013 (35.5)		47415 (36.0)	69715 (35.3)	
**Smoking status**						
Never	98557 (53.9)	82500 (56.2)	<0.001	72612 (55.1)	108445 (54.9)	<0.001
Previous	63259 (34.6)	49316 (33.6)		48404 (36.7)	64171 (32.5)	
Current	20084 (11.0)	14133 (9.6)		10170 (7.7)	24047 (12.2)	
**Drinking status**						
Never	7348 (4.0)	6751 (4.6)	<0.001	5656 (4.3)	8443 (4.3)	0.027
Previous	5942 (3.3)	5254 (3.6)		4345 (3.3)	6851 (3.5)	
Current	169042 (92.5)	134338 (91.6)		121560 (92.2)	181820 (92.0)	
**Coffee intake**	141661 (77.5)	114443 (78.0)	<0.001	102075 (77.4)	154029 (77.9)	<0.001
**Vitamin supplement**	53336 (29.2)	50031 (34.1)	<0.001	39937 (30.3)	63430 (32.1)	<0.001
**Mineral supplement**	71928 (39.4)	68372 (46.6)	<0.001	59410 (45.1)	80890 (40.9)	<0.001
**Sleep pattern**						
Poor	3770 (2.1)	2532 (1.7)	<0.001	2493 (1.9)	3809 (1.9)	<0.001
Intermediate	61078 (33.4)	45481 (31.0)		43117 (32.7)	63442 (32.1)	
Healthy	85475 (46.8)	73808 (50.3)		63197 (47.9)	96086 (48.6)	
**Cancer**	18799 (10.3)	17945 (12.2)	<0.001	16751 (12.7)	19993 (10.1)	<0.001
**CVD**	23865 (13.1)	18631 (12.7)	0.002	20480 (15.5)	22016 (11.1)	<0.001
**Depression**	10944 (6.0)	8592 (5.9)	0.329	7318 (5.6)	12218 (6.2)	<0.001
**Family history of kidney disorders**	**5 (0.0)**	**2 (0.0)**	**0.639**	**3 (0.0)**	**4 (0.0)**	**>0.99**
**Diabetes mellitus**	13454 (7.4)	11675 (8.0)	<0.001	10321 (7.8)	14808 (7.5)	0.014
**Course of diabetes**	**9.21 (13.0)**	**10.2 (13.4)**	**<0.001**	**5.00 (2.00,11.0)**	**5.00 (2.00,10.0)**	**0.034**
**Use of antidiabetic drug**	1391 (0.8)	1398 (1.0)	<0.001	987 (0.7)	1802 (0.9)	<0.001
**hypertension**	47301 (25.9)	40396 (27.5)	<0.001	41016 (31.1)	46681 (23.6)	<0.001
**Use of antihypertensive drug**	32903 (18.0)	28856 (19.7)	<0.001	30979 (23.5)	30780 (15.6)	<0.001
**High cholesterol**	4849 (2.7)	3713 (2.5)	0.177	4584 (3.5)	3978 (2.0)	<0.001
**Use of cholesterol lowing drug**	28479 (15.6)	22176 (15.1)	0.023	26293 (19.9)	24362 (12.3)	<0.001
**Use of ACEI/ARB**	**28 (0.0)**	**18 (0.0)**	**0.557**	**21 (0.0)**	**25 (0.0)**	**0.529**
**HDL cholesterol, mmol/L**	1.39 (0.355)	1.52 (0.388)	<0.001	1.44 (0.375)	1.45 (0.376)	<0.001
**LDL cholesterol, mmol/L**	3.56 (0.848)	3.58 (0.864)	<0.001	3.57 (0.871)	3.57 (0.844)	0.244
**Triglycerides, mmol/L**	1.82 (1.05)	1.63 (0.946)	<0.001	1.76 (0.955)	1.72 (1.05)	<0.001

**C-reactive protein, mg/L**	2.57 (4.23)	2.42 (4.11)	<0.001	2.58 (4.16)	2.46 (4.19)	<0.001
**Direct glucose, mmol/L**	5.06 (1.03)	5.10 (1.23)	<0.001	5.09 (1.02)	5.08 (1.19)	0.003
**Hba1c, mmol/mol**	35.7 (5.87)	35.9 (6.48)	<0.001	36.1 (5.46)	35.6 (6.57)	<0.001
**Urate, umol/L**	322 (77.2)	290 (75.2)	<0.001	327 (76.7)	295 (76.2)	<0.001
**eGFR, mL/min/1.73 m^2**	90.9 (12.2)	92.6 (11.6)	<0.001	79.7 (7.50)	99.6 (6.66)	<0.001
**UACR, mg/g**	6.31 (1.92)	16.5 (5.14)	<0.001	10.2 (6.04)	11.3 (6.43)	<0.001

Note: Data are presented as mean and standard variation (SD) for continuous variables, and as frequency and percentage (%) for categorical variables.

Abbreviations: BMI, body mass index; TDI, Townsend deprivation Index; MET, metabolic equivalent of task; MVPA, moderate-to-vigorous physical activity; CVD, cardiovascular disease; SBP, systolic blood pressure; HDL, high-density lipoprotein; LDL, low-density lipoprotein; HbA1c, glycated hemoglobin; eGFR, estimated glomerular filtration rate; UACR, urine albumin-creatinine ratio; Lp(a), lipoprotein a; ACEI, angiotensin converting enzyme inhibitor; ARB, angiotensin receptor blocker.

* Baseline UACR within the normal range was classified into low-normal (0 - 9.9 mg/g) and high-normal (10 - 29.9 mg/g) groups. Baseline eGFR within the normal range was classified into low (< 90 mL/min/1.73 m^2) and high (≥ 90 mL/min/1.73 m^2) group

STable 6. Associations between Lp(a), UACR and risks of chronic kidney disease using a parsimonious model

Variables	No. of cases / total participants	HR (95% CI), p-value
**Independent associations**		
**Lp(a)**		
Low group	4,642 / 258,388	Ref
High group	1,361 / 71,027	1.05 (0.98, 1.11), p = 0.178
**UACR**		
Low-normal group	3,247 / 182,740	Ref
High-normal group	2,756 / 146,675	1.22 (1.15, 1.29), p < 0.001
**Joint associations between baseline Lp(a) and UACR**		
Low Lp(a) & low-normal UACR	2,527 / 142,923	Ref
High Lp(a) & low-normal UACR	720 / 39,817	1.00 (0.91, 1.09), p = 0.965
Low Lp(a) & high-normal UACR	2,115 / 115,465	1.19 (1.12, 1.27), p < 0.001
High Lp(a) & high-normal UACR	641 / 31,210	1.32 (1.20, 1.45), p < 0.001

Abbreviations: UACR, urine albumin-creatinine ratio; Lp(a), lipoprotein a; HR, hazard ratio; CI, confidence interval.
* Baseline Lp(a) was categorized into low (< 75 nmol/L) and high (≥ 75 nmol/L) groups. Baseline UACR within the normal range was classified into low-normal (0 - 9.9 mg/g) and high-normal (10 - 29.9 mg/g) groups.
* Parsimonious model adjusted for age, sex, body mass index, comorbidities and use of drugs.

STable 7. Associations between Lp(a), UACR and risks of chronic kidney disease in participants with baseline eGFR over 90 mL/min/1.73 m^2

Variables	No. of cases / total participants	HR (95% CI), p-value
**Independent associations**		
**Lp(a)**		
Low group	740 / 155,074	Ref
High group	204 / 42,526	1.05 (0.88, 1.25), p = 0.613
**UACR**		
Low-normal group	479 / 103,707	Ref
High-normal group	465 / 93,893	1.07 (0.94, 1.23), p = 0.304
**Joint associations between baseline Lp(a) and UACR**		
Low Lp(a) & low-normal UACR	372 / 81,071	Ref
High Lp(a) & low-normal UACR	107 / 22,636	1.03 (0.83, 1.28), p = 0.788
Low Lp(a) & high-normal UACR	368 / 74,003	1.10 (0.95, 1.27), p = 0.187
High Lp(a) & high-normal UACR	97 / 19,890	1.12 (0.86, 1.35), p = 0.500

Abbreviations: UACR, urine albumin-creatinine ratio; Lp(a), lipoprotein a; HR, hazard ratio; CI, confidence interval.
* Baseline Lp(a) was categorized into low (< 75 nmol/L) and high (≥ 75 nmol/L) groups. Baseline UACR within the normal range was classified into low-normal (0 - 9.9 mg/g) and high-normal (10 - 29.9 mg/g) groups.
*Fully adjusted model adjusted for age, sex, body mass index, Townsend Deprivation Index, college degree, ethnicity, area, smoking and drinking status, regular intake of coffee, vitamin and mineral supplement, personal medical history of cancer, CVD, depression, diabetes, hypertension, high cholesterol, drugs for diabetes, hypertension, high cholesterol, SBP, HDL cholesterol, LDL cholesterol, TG, HbA1c, CRP, urate, eGFR.

STable 8. Associations between Lp(a), UACR and risks of chronic kidney disease after removal of several disease occurrences during follow up of the study

**Variables**	**Removal of diabetes mellitus occurrence**		**Removal of hypertension occurrence**		**Removal of obesity occurrence**	
	**No. of cases / total participants**	**HR (95% CI), p-value**	**No. of cases / total participants**	**HR (95% CI), p-value**	**No. of cases / total participants**	**HR (95% CI), p-value**
**Joint associations between baseline Lp(a) and UACR**						
Low Lp(a) & low-normal UACR	1,797 / 128,242	Ref	1,241 / 113,541	Ref	2,114 / 133,537	Ref
High Lp(a) & low-normal UACR	509 / 35,671	0.98 (0.88, 1.10), p = 0.729	339 / 31,346	0.94 (0.82, 1.08), p = 0.408	594 / 37,106	0.98 (0.88, 1.08), p = 0.660
Low Lp(a) & high-normal UACR	1,460 /102,538	1.17 (1.08, 1.27), p < 0.001	1,045 / 90,783	1.21 (1.10, 1.33), p < 0.001	1,829 / 109,492	1.17 (1.08, 1.25), p < 0.001
High Lp(a) & high-normal UACR	439/27,737	1.29 (1.14, 1.45), p < 0.001	301 / 24,406	1.28 (1.11, 1.48), p < 0.001	561 / 29,579	1.32 (1.19, 1.48), p < 0.001

Abbreviations: UACR, urine albumin-creatinine ratio; Lp(a), lipoprotein a; HR, hazard ratio; CI, confidence interval; CKD, chronic kidney disease; ESRD, end-stage renal disease.
* Baseline Lp(a) was categorized into low (< 75 nmol/L) and high (≥ 75 nmol/L) groups. Baseline UACR within the normal range was classified into low-normal (0 - 9.9 mg/g) and high-normal (10 - 29.9 mg/g) groups.
*Models adjusted for sex, age, body mass index, Townsend Deprivation Index, college degree, ethnicity, area, smoking and drinking status, regular intake of coffee, vitamin and mineral supplement, personal medical history of cancer, CVD, depression, diabetes, hypertension, high cholesterol, drugs for diabetes, hypertension, high cholesterol, SBP, HDL cholesterol, LDL cholesterol, TG, HbA1c, CRP, urate, eGFR.

STable 9. Associations between Lp(a), UACR and risks of secondary outcomes

Variables	Moderate CKD (stage 3)		Severe CKD (stage 4)		CKD stage 5 and ESRD	
	**No. of cases / total participants**	**HR (95% CI), p-value**	**No. of cases / total participants**	**HR (95% CI), p-value**	**No. of cases / total participants**	**HR (95% CI), p-value**
**Independent associations**						
**Lp(a)**						
Low group	4,349 / 258,388	Ref	302 / 258,388	Ref	195 / 258,388	Ref
High group	1,266 / 71,027	1.04 (0.97, 1.12), p = 0.304	91 / 71,027	1.16 (0.89, 1.51), p = 0.271	57 / 71,027	1.15 (0.82, 1.61), p = 0.419
**UACR**						
Low-normal group	3,041 / 182,740	Ref	199 / 182,740	Ref	128 / 182,740	Ref
High-normal group	2,574 / 146,675	1.19 (1.12, 1.26), p < 0.001	192 / 146,675	1.49 (1.18, 1.89), p < 0.001	124 / 146,675	1.45 (1.08, 1.94), p < 0.001
**Joint associations between baseline Lp(a) and UACR**						
Low Lp(a) & low-normal UACR	2,366 / 142,923	Ref	156 / 142,923	Ref	99 / 142,923	Ref
High Lp(a) & low-normal UACR	675 / 39,817	0.99 (0.90, 1.08), p = 0.645	43 / 39,817	1.05 (0.72, 1.53), p = 0.810	29 / 39,817	1.22 (0.77, 1.92), p = 0.393
Low Lp(a) & high-normal UACR	1,983 / 115,465	1.15 (1.08, 1.24), p < 0.001	146 / 115,465	1.42 (1.09, 1.86), p = 0.009	96 / 115,465	1.49 (1.07, 2.08), p = 0.019
High Lp(a) & high-normal UACR	591 / 31,210	1.29 (1.16, 1.43), p < 0.001	48 / 31,210	1.83 (1.26, 2.66), p < 0.001	28 / 31,210	1.60 (0.97, 2.64), p = 0.067

Abbreviations: UACR, urine albumin-creatinine ratio; Lp(a), lipoprotein a; HR, hazard ratio; CI, confidence interval; CKD, chronic kidney disease; ESRD, end-stage renal disease.

* Baseline Lp(a) was categorized into low (< 75 nmol/L) and high (≥ 75 nmol/L) groups. Baseline UACR within the normal range was classified into low-normal (0 - 9.9 mg/g) and high-normal (10 - 29.9 mg/g) groups.

*Models adjusted for sex, age, body mass index, Townsend Deprivation Index, college degree, ethnicity, area, smoking and drinking status, regular intake of coffee, vitamin and mineral supplement, personal medical history of cancer, CVD, depression, diabetes, hypertension, high cholesterol, drugs for diabetes, hypertension, high cholesterol, SBP, HDL cholesterol, LDL cholesterol, TG, HbA1c, CRP, urate, eGFR.

SFigure 1. Flowchart for the study design

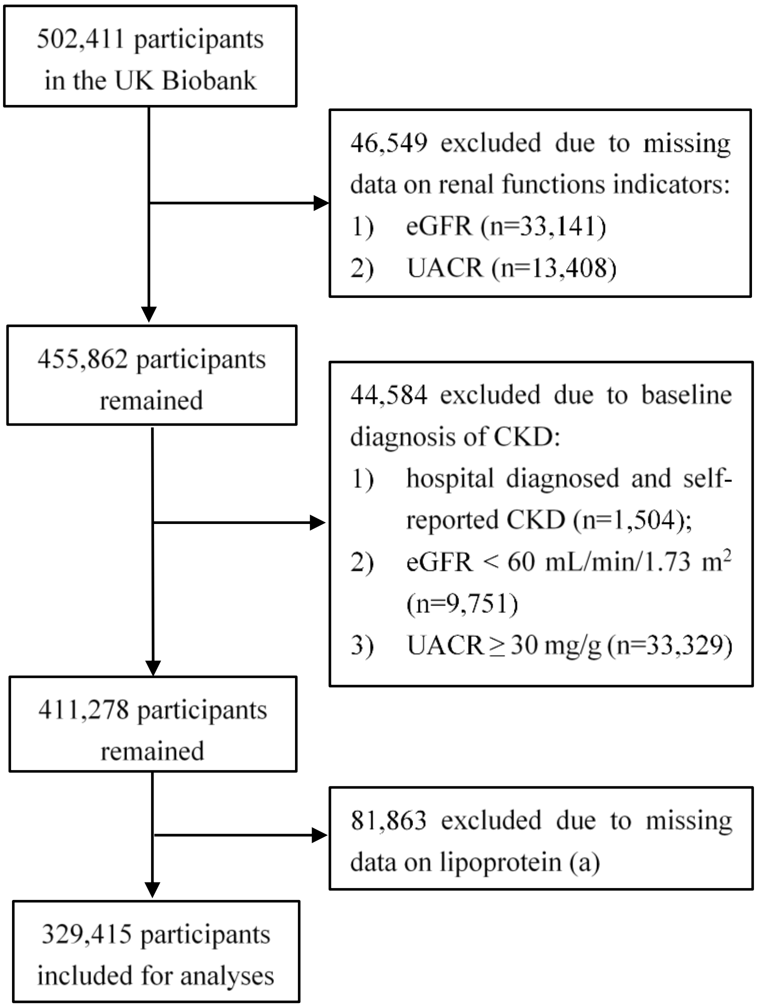

Supplement: Multimedia Appendix 1 [file publichealth_v10i1e50415_app1.pdf]
